# Ethylene-Induced Hydrogen Sulfide Negatively Regulates Ethylene Biosynthesis by Persulfidation of ACO in Tomato Under Osmotic Stress

**DOI:** 10.3389/fpls.2018.01517

**Published:** 2018-10-17

**Authors:** Honglei Jia, Sisi Chen, Dan Liu, Johannes Liesche, Cong Shi, Juan Wang, Meijuan Ren, Xiaofeng Wang, Jun Yang, Wei Shi, Jisheng Li

**Affiliations:** ^1^Biomass Energy Center for Arid and Semi-Arid Lands, College of Life Sciences, Northwest A&F University, Yangling, China; ^2^School of Environmental Science and Engineering, Shaanxi University of Science and Technology, Xi’an, China; ^3^Northwest A&F University Life Science Research Core Services, Northwest A&F University, Yangling, China

**Keywords:** hydrogen sulfide, ethylene, persulfidation, feedback regulation, tomato, LeACOs, abiotic stress

## Abstract

A number of recent studies identified hydrogen sulfide (H_2_S) as an important signal in plant development and adaptation to environmental stress. H_2_S has been proven to participate in ethylene-induced stomatal closure, but how the signaling pathways of H_2_S and ethylene interact is still unclear. Here, we reveal how H_2_S controls the feedback-regulation of ethylene biosynthesis in tomato (*Solanum lycopersicum*) under osmotic stress. We found that ethylene induced the production of H_2_S in guard cells. The supply of hypotaurine (HT; a H_2_S scavenger) or DL-pro-pargylglycine (PAG; a synthetic inhibitor of H_2_S) removed the effect of ethylene or osmotic stress on stomatal closure. This suggests that ethylene-induced H_2_S is a downstream component of osmotic stress signaling, which is required for ethylene-induced stomatal closure under osmotic stress. We further found that H_2_S inhibited ethylene synthesis through inhibiting the activity of 1-aminocyclopropane-1-carboxylic acid (ACC) oxidases (ACOs) by persulfidation. A modified biotin-switch method (MBST) showed that H_2_S can induce persulfidation of LeACO1 and LeACO2 in a dose-dependent manner, and that persulfidation inhibits the activity of LeACO1 and LeACO2. We also found that LeACO1 is persulfidated at cysteine 60. These data suggested that ethylene-induced H_2_S negatively regulates ethylene biosynthesis by persulfidation of LeACOs. In addition, H_2_S was also found to inhibit the expression of *LeACO* genes. The results provide insight on the general mode of action of H_2_S and contribute to a better understanding of a plant’s response to osmotic stress.

## Introduction

Hydrogen sulfide (H_2_S) is recognized as the third endogenous gasotransmitter, following the discovery of nitric oxide and carbon monoxide (Tan et al., 2010). In plants, cysteine (Cys) metabolism is closely related to H_2_S generation. Cys desulfhydrases (CDes) contribute to H_2_S generation (Papenbrock et al., 2007). L-Cys desulfhydrase (LCD) and D-Cys desulfhydrase (D-CDES) degrade Cys to H_2_S, pyruvate, and ammonia and are responsible for the release of H_2_S into the cell (Kopriva, 2006). H_2_S signaling has been implicated in the regulation of photosynthesis, immunity, cell senescence, root growth, and stomatal closure (Wang et al., 2002; Xie et al., 2014; Jia et al., 2015). H_2_S has been similarly recognized as a major signaling compound, involved in stress responses to osmotic stress, high salinity, drought, heavy metals, and oxidative stress (Christou et al., 2013; Li et al., 2014; Jia et al., 2016; Fang et al., 2016; Guo et al., 2017). Interestingly, H_2_S seems to generally act via the modulation of hormone action, namely of ethylene, auxin, and abscisic acid (Hou et al., 2013; Scuffi et al., 2014; Jia et al., 2015). Not known, so far, is the molecular mechanism of how H_2_S can affect hormone action.

Here, the mode of action of H_2_S on ethylene signaling is investigated in tomato. Ethylene plays critical roles in fruit maturation, plant growth, and adaptation to environmental challenges (Lacey and Binder, 2014). Environmental stimuli, such as drought and mechanical injury, increase ethylene concentration in plant cells through increased ethylene biosynthesis (Groen and Whiteman, 2014). The biosynthesis of ethylene is mediated by 1-aminocyclopropane-1-carboxylic acid (ACC) synthases (ACSs) and ACC oxidases (ACOs). ACOs have been identified as the enzymes catalyzing the rate-limiting of ethylene biosynthesis (Bleecker and Kende, 2000). ACOs are encoded by a multi-gene family in plants. It is reported that *Arabidopsis thaliana* has five *AtACO* genes, and that *Solanum lycopersicum* has six *LeACO* genes (Kende, 1993). Expression analysis revealed that *ACOs* show a high tissue specificity, with *LeACO1* and *LeACO2* expressed in guard cells (Spanu et al., 1991). Many reports show that the silencing of *ACO* genes or the inhibition ACO activity reduces ethylene content in plant cells (Chao et al., 2013; Satoh and Kosugi, 2017).

The biosynthesis of ethylene is regulated by a feedback mechanism and by other signaling molecules. For example, auxin was shown to induce ethylene synthesis via the transcription of *ACO*s in *Oryza sativa* (Argueso et al., 2007) and nitric oxide was shown to decrease ethylene content through inhibiting ethylene synthesis enzyme activity (Zhu et al., 2006). Recently, H_2_S has been implicated in the regulation of ethylene action (Liu et al., 2011). It was reported that H_2_S weakens the effect of ethylene on banana fruit ripening (Ge et al., 2017) and inhibits ethylene-induced cell senescence in kiwifruit (Gao et al., 2013). However, the mechanism of H_2_S regulating ethylene biosynthesis is unknown.

The working hypothesis is that it acts by persulfidation, which is the post-translational modification of Cys residues to form a persulfide group (cysteine -SH groups are converted to -SSH). Recent evidence indicates that persulfidation by endogenous H_2_S regulates the function of certain proteins, such as ascorbate peroxidase1 and glyceraldehyde 3-phosphate dehydrogenase (Aroca et al., 2015, 2017b). In addition, persulfidation was demonstrated *in vivo* in *Arabidopsis*, and that AtACO1 could be among the many proteins that can be modified in this way (Aroca et al., 2017a). Therefore, we tested here if tomato ACOs are subject to H_2_S-induced persulfidation and if this is the mechanism by which H_2_S modulates ethylene signaling in response to osmotic stress. Experiments were carried out on tomato because its sensitivity to osmotic stress has been recognized as a major challenge to increase the production efficiency of this important crop.

## Materials and Methods

### Plant Material and Chemical Treatments

Tomato (*S. lycopersicum*) seeds (Liger) were purchased from the Tomato Research and Development Center of Xinjiang Shihezi Vegetable Research Institute. The seeds were sterilized with 3% sodium hypochlorite for 15 min, then washed with ddH_2_O for three times. The seeds were sown in a tray with vermiculite and cultivated in a climate chamber [16 h/8 h, light/dark photoperiod, 60% relative humidity, temperature of 26°C (day)/20°C (night)]. After the second leaf emerged, plants were moved to hydroponics cultures with 1/4 Hoagland’s nutrient solution (pH = 6.5). After one-week adaptation, uniform individuals were selected and transplanted in the hydroponic pots with 1/2 Hoagland’s nutrient solution. After another week, the seedings were used for different treatments and analyses.

Experiment 1: Two-week-old tomato seedlings were supplied with between 0 and 25% PEG6000 (PEG), 200 μM NaHS and 1.0 g/L ethephon for 3 days, and then the phenotype was observed. Two-week-old tomato seedlings were supplied with between 0 and 25% PEG, 200 μM NaHS and 1.0 g/L ethephon for 6 h, and then the stomatal aperture and relative water content (RWC) was measured.

Experiment 2: Two-week-old tomato seedlings were supplied with 25% PEG, 200 μM NaHS, 200 μM H_2_S scavenger hypotaurine (HT; Shanghai Aladdin Biochemical Technologies), 2 μM the H_2_S inhibitor DL-propargylglycine (PAG; Shanghai Aladdin Biochemical Technologies), 1.0 g/L Ethephon and 200 μM the ethylene biosynthesis inhibitor aminoethoxyvinylglycine (AVG; Shanghai Aladdin Biochemical Technologies) for 6 h, and then endogenous endogenous H_2_S content was measured.

Experiment 3: Two-week-old tomato seedlings were supplied with 25 % PEG and 200 μM NaHS for 6–72 h, and then endogenous ethylene content and ACO activity was measured.

Experiment 4: Two-week-old tomato seedlings were supplied with 25% PEG, 200 μM NaHS and 1.0 g/L Ethephon for 3 h or 24 h, and then relative expression of *LeACO1* and *LeACO2* was measured.

### Relative Water Content Assay

The sample was dried at 80°C for 48 h. RWC = [(FW-DW)/FW] × 100%.

### Stomatal Aperture Measurements

Stomatal aperture was performed as described by Hou et al. (2013). The epidermis was stripped from tomato seedlings and immediately incubated in opening buffer (0.1 mM CaCl_2_, 50 mM KCl and 10 mM MES, pH = 6.1) under light conditions (200 μM/m^2^/s) for 3 h. The epidermal peels with pre-opened stomatal were subsequently maintained in the same opening buffer and exposed to different treatments. After 90 min, stomata were photographed using a light microscope. The stomatal aperture width was measured using ImageJ analysis software.

### Ethylene Content Assay

Ethylene content quantification was performed as described by Li et al. (2014). Two-week-old tomato seedlings were supplied with 25% PEG and 200 μM NaHS for 0–72 h, every treatment contains six samples (0, 6, 12, 24, 36, and 72 h). Each sample (2 g) was placed in 10-ml gas tight glass vessels and incubated at room temperature for 10 h. After incubated for 10 h in the glass vessels, the sample of gas was used for the measurement of ethylene. One milliliter sample of gas was removed and analyzed with a flame ionizing gas chromatograph (model 3700, Varian Medical Systems, Palo Alto, CA, United States) equipped with Porapak Q column (80–100 mesh, 1 m × 3.2 mm). Oven, injector, and detector temperatures were 50, 150, and 200°C, respectively.

### ACO Activity Assay

1-aminocyclopropane-1-carboxylic acid (ACC) oxidases activity was determined using protein extracts obtained from tomato seedling leaves and the purified recombinant LeACO1 and LeACO2 proteins. Protein extraction buffer was 50 mM Tris pH = 7.4. The activity of ACO was determined according to Madhaiyan et al. (2007). Recombinant proteins were pretreated with 200 μm NaHS and 10 mM DTT at 4°C for 20 min, and then enzyme activity was analyzed. The enzyme activity was assayed at 30°C for 15 min. 1.5 mL of protein supernatant was incubated with 50 μM FeSO4, 1 mM ACC, and 5% (v/v) CO_2_, and then the quantity of released ethylene was determined. The protein concentrations were estimated by Coomassie brilliant blue staining and using bovine serum albumin as a reference protein.

### H_2_S Content Assay in Guard Cells and Whole Leaves

The specific H_2_S fluorescent probe 7-azido-4-methylcoumarin (AzMC; Sigma-Aldrich) was used for the analysis of H_2_S content in guard cells. After treatments, leaves were incubated in 20 mM HEPES NaOH buffer (pH 7.5) containing 20 μM of the probe for 30 min in darkness (25°C). Afterward, the leaves were washed three times (15 min each time) with fresh HEPES buffer, and guard cells were observed under a spinning-disc confocal microscope (Andor, United Kingdom, Revolution-WD). Z-stack were recorded to obtain information from the entire guard cell volume. ImageJ was used for the analysis of fluorescence intensity.

The content of endogenous H_2_S was measured according to Chen et al. (2016). The leaves were ground and extracted in 10 mL of phosphate buffered saline (pH 6.8, 50 mM) containing 0.1 mM EDTA and 0.2 mM ascorbic acid. The homogenate was mixed in a test tube containing 100 mM phosphate buffered saline (pH 7.4), 10 mM L-cysteine and 2 mM phosphopyridoxal at room temperature, and the released H_2_S was absorbed in a zinc acetate trap. The trap consisted of a small glass tube containing 3 mL of 0.5% zinc acetate that was fixed to the bottom of the reaction bottle. After a 30 min reaction, 0.3 mL of 20 mM dimethyl-p-phenylenediamine was dissolved in 7.2 mM HCl and was added to the trap. This was followed by injection of 0.3 mL of 30 mM ferric ammonium sulfate in 1.2 mL HCl. After incubation for 15 min at room temperature, the amount of H_2_S in the zinc acetate trap was determined colorimetrically at 667 nm. A calibration curve was made according to the above method and H_2_S content in seedlings was expressed as nmol g^−1^ DW.

### Recombinant Protein Expression and Site-Directed Mutagenesis

For *in vitro* protein expression, the coding regions of LeACO1 (GI: 902763266) and LeACO2 (GI: 1049480165) were amplified and inserted in-frame into the plasmid pET30a. The recombinant his-tagged proteins were purified from *Escherichia coli* BL21 cells using Amylose Resin (New England Biolabs) following the manufacturer’s manual. Site-directed mutagenesis was carried out using the Quickchange II Site-Directed Mutagenesis Kit (Stratagene) following the manufacturer’s manual. The primers used in this study are listed in **Supplementary Table S1**.

### Immunochemical Detection of Persulfidated LeACOs

Persulfidated proteins were detected using a modified biotin-switch method (MBST, Mustafa et al., 2009). The purified recombinant LeACO1 and LeACO2 proteins were treated with 50 to 400 μM NaHS to increase the concentration of persulfidated protein or with 10 mM DTT to reduce all of the disulfide bonds; both treatments were carried out at 4°C for 20 min. NaHS was removed using Micro BioSpinP6columns (BioRad). The proteins were blocked with 20 mM methyl methanethiosulfonate (MMTS), and the persulfidated cysteines were labeled by 4 mM biotin in the presence of N- [6-(biotinamido) hexyl]-39-(2′-pyridyldithio)-propionamide (biotin-HPDP). Persulfidated proteins were detected by immunoblot using an anti-biotin antibody (Sino Biological). The total amount of LeACO1 and LeACO2 proteins was determined using an anti-his antibody (Sino Biological). Persulfidated proteins were detected by anti-biotin antibody (YEASEN). Antibodies working concentration: anti-his antibody is 10 mU/ml, anti-biotin antibody is 0.5 μg/ul. Gray analysis of ImageJ software ^1^ was used for quantification persulfidated levels.

### Identification of Persulfidated Cys Residues

Purified recombinant protein was separated using non-reducing SDS–PAGE on 12% (w/v) polyacrylamide gels, and the bands corresponding to LeACO1 was excised manually from Coomassie-stained gels and then deposited in 96-well plates. Samples were alkylated by 50 mM iodoacetamide (Sigma-Aldrich) for 40 min in darkness. Disulfide bonds were restored by treatment with 10 mM DTT at 37°C for 3 h. Samples were digested by Trypsin (Promega) at a mass ratio of 1:50 in 37°C for 16 h. Digested peptides were dissolved in sample solution (0.1% formic acid and 2% acetonitrile), and centrifuged at 13200 rpm at 4°C for 10 min. The supernatant was analyzed by mass spectrometry (Thermo Scientific Q Exactive) using a chromatographic column of 300 μm i.d. × 5mm packed with Acclaim PepMap RPLC C18, 5 μm, 100 Å and a column of 75 μm i.d. × 150 mm, packed with Acclaim PepMap RPLC C18, 3 μm, 100 Å. Mobile phase A: 0.1% methanoic acid and 2% ACN. Mobile phase B: 0.1% methanoic acid and 80% ACN. Flow rate: 300 nL min^−1^. Analysis time: 78 min. Data was analyzed by Maxquant software referencing the Uniprot database. Peptide mass tolerance was set to 10 ppm. 0.6 Da fragment masses and two miss cleavages were adopted in this experiment. Peptides score ≥ 20 were considered correctly identified.

### RNA Isolation and qRT-PCR

Leaves of tomato seedling were harvested to extract total RNA for real-time PCR. Total RNA was extracted using RNAprep pure plant kit (Takara bio, Beijing) and treated with RNase free DNase (Takara bio, Beijing). The total RNA was reverse-transcribed into first-strand cDNA using PrimeScriptTM Reverse Transcriptase and Oligo (dT)15 primer following the manufacturer’s instructions. The samples were amplified using SYBR Green I (SYBR^®^ Premix Ex TaqTM Kit, Takara bio, Beijing), Real-time system used the BIO-RAD CFXF96. The housekeeping gene *Ubi3* was used as an internal control. The PCR amplifications for each gene were performed in triplicate. The results were analyzed by Rotor-Gene Real-Time Analysis Software 6.1 (Build 81). The primers used in this study are listed in **Supplementary Table S1**.

### Statistical Analysis

Each experiment was repeated at least three times with three replications per experiment. Values were expressed as means ± SE. For all experiments, the overall data were statistically analyzed in SPSS version 17.0 (SPSS). Duncan’s multiple range tests were used. The statistical analysis of two groups was performed using Student’s *t*-test. In all cases, the confidence coefficient was set at 0.05.

## Results

### H_2_S and Ethylene Induce Stomatal Closure and Water Retention Under Osmotic Stress

**FIGURE 1:**
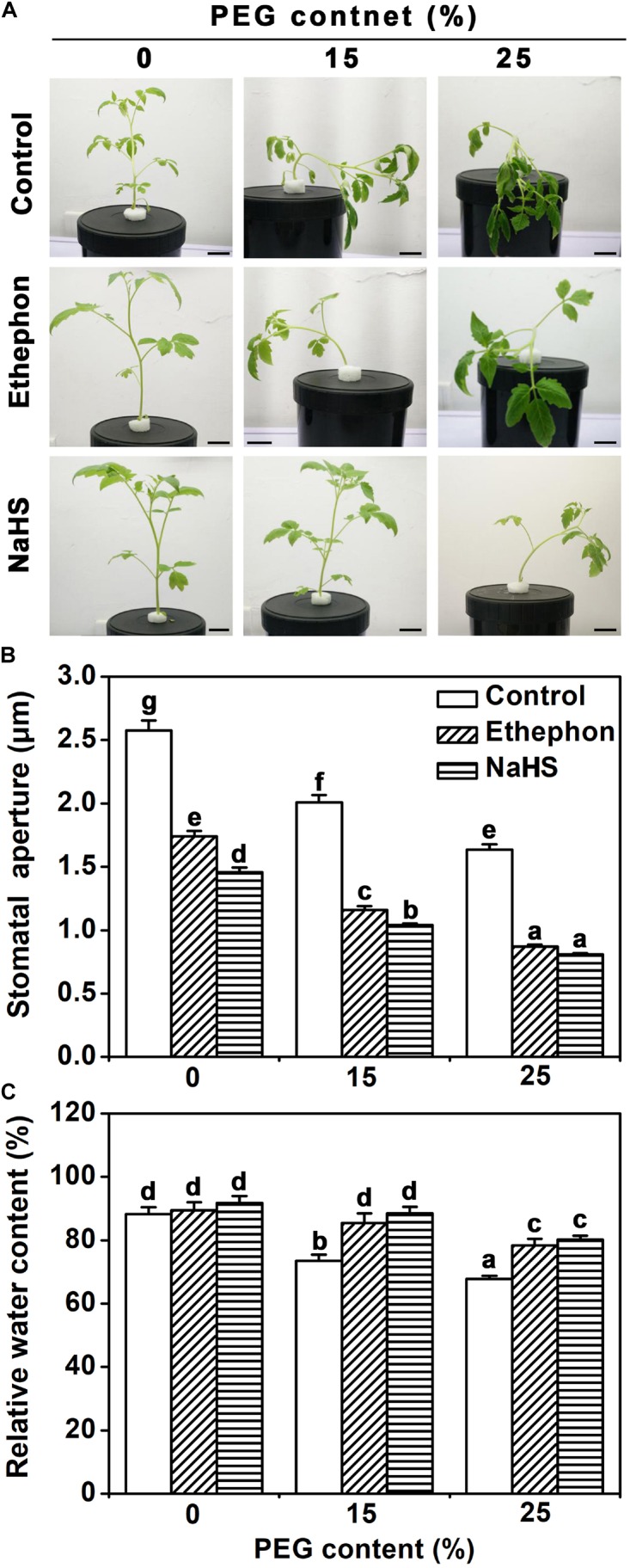
Biological phenotype assays. **(A)** Photographs of tomato seedlings. Two-week-old tomato seedlings were supplied with between 0 and 25% PEG, 200 μM NaHS and 1.0 g/L ethephon for 3 days, and then the phenotype was observed (Bar = 2 cm). **(B)** Two-week-old tomato seedlings were supplied with between 0 and 25% PEG, 200 μM NaHS and 1.0 g/L ethephon for 6 h, and then the stomatal aperture was measured. The data are mean values ± SE (*n* = 50). **(C)** Two-week-old tomato seedlings were supplied with between 0 and 25% PEG, 200 μM NaHS and 1.0 g/L ethephon for 6 h, and then relative water content (RWC) was measured. Mean values ± SE are calculated from three replicates. Within each set of experiments, bars with different letters are significant different (Duncan’s multiple range tests, *P* < 0.05).

Exposure of tomato seedlings to 15 or 25% PEG resulted in softening of shoots and severe wilting of leaves (**Figure 1A**). Treatment with the H_2_S precursor NaHS or the ethylene donor ethephon partly prevented this PEG-response (**Figure 1A**). Plants treated with NaHS or ethephon showed a higher rate of stomatal closure compared to control plants (**Figure 1B**). While PEG treatment caused the relative water content of leaves to decrease in control plants, plants treated with NaHS or ethephon did not show any reduction in response to 15% PEG, and relative water content decreased slightly in response to 25% PEG (**Figure 1C**). This data suggested that H_2_S and ethylene influence the osmotic stress response via regulation of stomata closure.

### Ethylene Induces Accumulation of H_2_S

**FIGURE 2:**
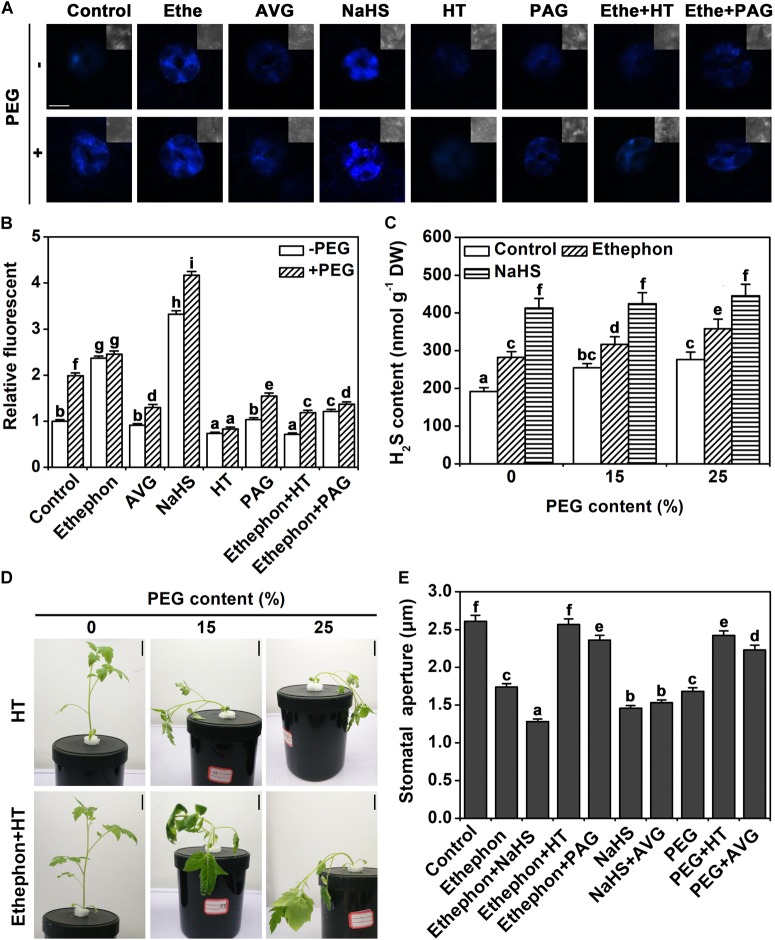
Analysis of endogenous H_2_S content. **(A)** H_2_S AzMC fluorescence in guard cells (bar = 10 μm). Two-week-old tomato seedlings were supplied with 25% PEG, 200 μM NaHS, 200 μM HT, 2 μM PAG, 1.0 g/L Ethephon, and 200 μM AVG for 6 h, and then endogenous H_2_S content was measured in guard cells. **(B)** Quantization of H_2_S AzMC fluorescence density. The data are mean values ± SE (*n* = 20). **(C)** H_2_S content in leaves. Two-week-old tomato seedlings were supplied with 0–25% PEG, 200 μM NaHS, and 1.0 for 6 h, and then endogenous H_2_S content was measured in leaves. **(D)** Photographs of tomato seedlings. Two-week-old tomato seedlings were supplied with between 0 and 25% PEG, 200 μM HT and 1.0 g/L ethephon for 3 days, and then the phenotype was observed (Bar = 2 cm). **(E)** Stomatal aperture assays. Two-week-old tomato seedlings were supplied with 25% PEG, 200 μM NaHS, 200 μM HT, 2 μM PAG, 1.0 g/L Ethephon and 200 μM AVG for 6 h, and then stomatal aperture was measured. Mean values ± SE are calculated from three replicates. Within each set of experiments, bars with different letters are significant different (Duncan’s multiple range tests, *P* < 0.05).

In a next step, we tested how the H_2_S content in guard cells changes in response to the different treatments using the fluorescent probe 7-azido-4-methylcoumarin (AzMC). Ethephon and osmotic stress both increased the endogenous H_2_S content (**Figures 2A,B**). Aminoethoxyvinylglycine (AVG, the ethylene biosynthesis inhibitor) did not alter the H_2_S content in control condition, but it inhibited the accumulation of H_2_S under osmotic stress conditions (**Figures 2A,B**). Treatment with hypotaurine (HT, a H_2_S scavenger), or DL-pro-pargylglycine (PAG, a synthetic inhibitor of H_2_S) prevented the accumulation of H_2_S under osmotic stress. HT or PAG treatment also inhibited ethylene-induced H_2_S accumulation (**Figures 2A,B**). In addition, treatment with NaHS significantly enhanced H_2_S content in control condition or osmotic stress (**Figures 2A,B**). H_2_S contents also were analyzed in the whole leaf in the presence of PEG at various concentrations. Data showed that the supply of 15 and 25% PEG induced H_2_S accumulation (**Figure 2C**). Ethephon also increased H_2_S content in both control and stress conditions in whole leaves (**Figure 2C**).

### H_2_S Is Involved in Ethylene-Induced Stomatal Closure

The wilting of the seedling was aggravated by HT under osmotic stress (**Figure 2D**). In addition, treatment with HT removed the effect of ethylene on osmotic stress (**Figure 2D**). To further investigate the relationship between H_2_S and ethylene on stomatal closure, a pharmacological experiment was performed. As indicated above, H_2_S, ethylene, and osmotic stress signals induced stomatal closure. The supply of HT or PAG countered the effect of ethylene or osmotic stress on stomatal closure (**Figure 2E**). However, the supply of AVG did not change the effect of H_2_S signal (**Figure 2E**). This result indicates that H_2_S may be an essential part of the signaling network of osmotic stress-induced stomatal closure.

### H_2_S Inhibits Ethylene Content in Leaves

**FIGURE 3:**
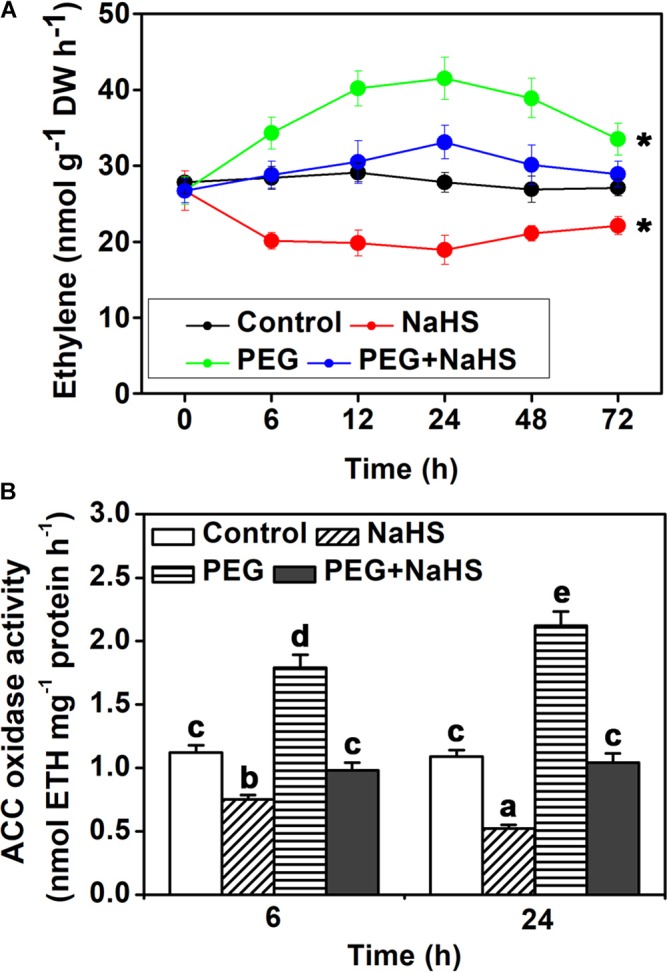
Analysis of endogenous ethylene content and ACO activity. **(A)** Time curve of ethylene content. **(B)** ACO activity. Two-week-old tomato seedlings were supplied with 25% PEG and 200 μM NaHS for 6–72 h, and then endogenous ethylene content and ACO activity were measured in leaves. Mean values ± SE are calculated from three replicates. Within each set of experiments, bars with different letters are significant different (Student’s *t*-test, ^∗^*P* < 0.05; Duncan’s multiple range tests, *P* < 0.05).

In addition to H_2_S content, also concentrations of ethylene were measured. As shown in **Figure 3A**, under osmotic stress, ethylene content increased after 6 h and reached the maximum after 24 h, and then decreased after 48 h. NaHS treatment significantly decreased ethylene content under control condition, and it also weakened the increase of osmotic stress-induced ethylene content (**Figure 3A**). In line with the change of ethylene content, ACC oxidase activity was increased by osmotic stress at the 6 h and 24 h time points (**Figure 3B**). NaHS treatment inhibited the activity of ACC oxidase under control and osmotic stress conditions (**Figure 3B**). These results suggest that H_2_S may inhibit ethylene biosynthesis through negatively regulating ACC oxidase activity.

### H_2_S-Induced Persulfidation of LeACO

**FIGURE 4:**
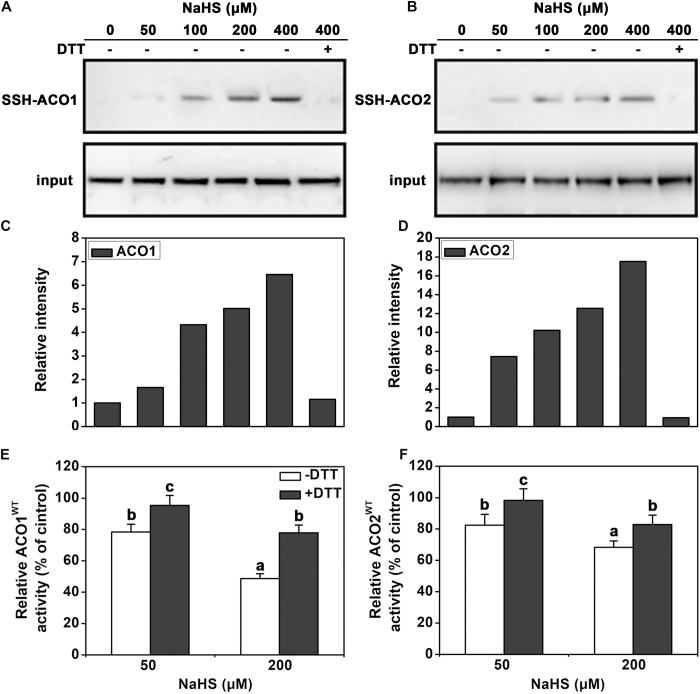
Immunoblot and activity analysis of recombinant LeACO1 and LeACO2 proteins. **(A)** NaHS-induced persulfidation of LeACO1 was detected using a MBST. **(B)** Quantification of persulfidation levels that are shown in **(A)**. **(C)** NaHS-induced persulfidation of LeACO2 was detected using a MBST. **(D)** Quantification of persulfidation levels that are shown in **(C)**. Input is anti-his signal, which indicate the total protein amount. Persulfidated proteins were labeled with biotin and analyzed using an anti-biotin antibody. Total proteins were analyzed using an anti-his antibody. Effect of NaHS-induced persulfidation on LeACO1 **(E)** and LeACO2 **(F)** activity. Recombinant proteins were pretreated with NaHS and NaHS plus DTT (10 mM) for 20 min, and then the immunoblot analysis carried out. Mean values ± SE are calculated from three replicates. Within each set of experiments, bars with different letters are significant different (Duncan’s multiple range tests, *P* < 0.05).

To determine how the activity of ACC oxidases is regulated by H_2_S, persulfidation assays were performed. The two ACC oxidases of tomato leaves, LeACO1 and LeACO2, were heterologously expressed and purified. Results indicated that NaHS induced persulfidation of LeACO1 and LeACO2 in a dose-dependent manner and that persulfidation was abolished by the application of DTT (**Figures 4A–D**). This shows that both proteins can be persulfidated by H_2_S *in vitro*.

To confirm the effect of persulfidation on ACC oxidase activity, the purified recombinant proteins were pretreated with NaHS or NaHS + DTT. NaHS pretreatment inhibited LeACO1 and LeACO2 activities in a dose-dependent manner. The addition of DTT canceled out the effect of NaHS (**Figures 4E,F**). These data suggested that H_2_S negatively regulates the activity of ACC oxidase activity by persulfidation *in vitro*.

### The Location of Persulfidation Sites in LeACO1

**FIGURE 5:**
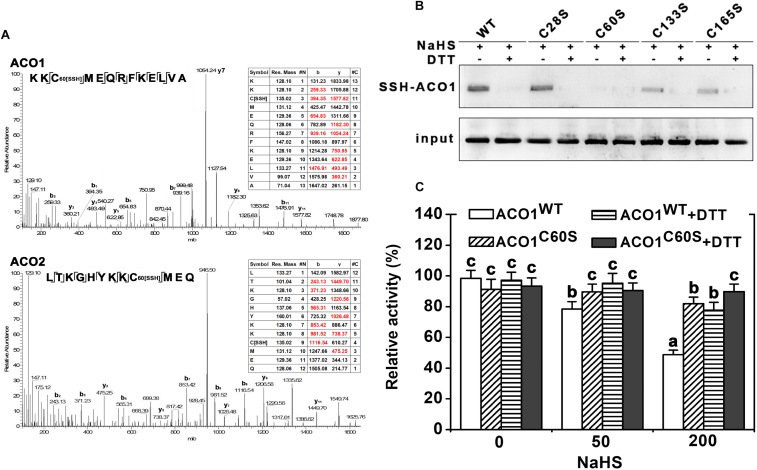
LC-MS/MS analysis of the tryptic peptide containing Cys60 of LeACO1 and LeACO2 recombinant protein **(A)**. The table inside the spectrum contains the predicted ion types for the modified peptide, and the ions detected in the spectrum are highlighted in red. A 32 D mass shift in Cys60 were included in the analysis of data by Maxquant software. Immunoblot analysis of recombinant LeACO1 mutation proteins **(B)**. NaHS-induced persulfidation of LeACO1 mutation was detected using a MBST. Recombinant proteins were pretreated with NaHS (200 μM) and NaHS plus DTT (10 mM) for 20 min, and then the immunoblot analysis carried out. Persulfidated proteins were labeled with biotin and analyzed using an anti-biotin antibody. Input is anti-his signal, which indicate the total protein amount. Analysis of recombinant LeACO1^C60S^ mutation protein activity **(C)**. Recombinant proteins were pretreated with NaHS and NaHS plus 10 mM DTT for 20 min, and then enzyme activity was analyzed. Mean values ± SE were calculated from three replicates. Within each set of experiments, bars with different letters are significant different (Duncan’s multiple range tests, *P* < 0.05).

Four cysteine residues in LeACO1 and LeACO2 are putative target sites of persulfidation. We carried out liquid chromatography (LC)-tandem mass spectrometry (MS/MS) analysis on the recombinant LeACO1 protein. The mass of Cys60 increased, which suggested that this residue had gained a sulfhydryl modification (**Figure 5A**). To determine whether the Cys60 residue is required for NaHS-mediated persulfidation, we mutated each cysteine (C) to serine (S) separately in LeACO1 and tested the effect of NaHS on the modified proteins. Of the four mutations, only the Cys60Ser (C60S) mutation eliminated persulfidation by NaHS (**Figure 5B**). To confirm that LeACO1 is persulfidated at Cys60 in the presence of the H_2_S donor, the recombinant LeACO1^C60S^ mutation protein was purified. The activity of LeACO1^C60S^ was similar with LeACO1^WT^ *in vitro*. However, compared with LeACO1^WT^, LeACO1^C60S^ was insensitive to NaHS treatment (**Figure 5C**). In addition, DTT removed the effect of NaHS (**Figure 5C**).

### H_2_S Inhibited the Expression of *LeACO1* and *LeACO2* Genes Under Osmotic Stress

**FIGURE 6:**
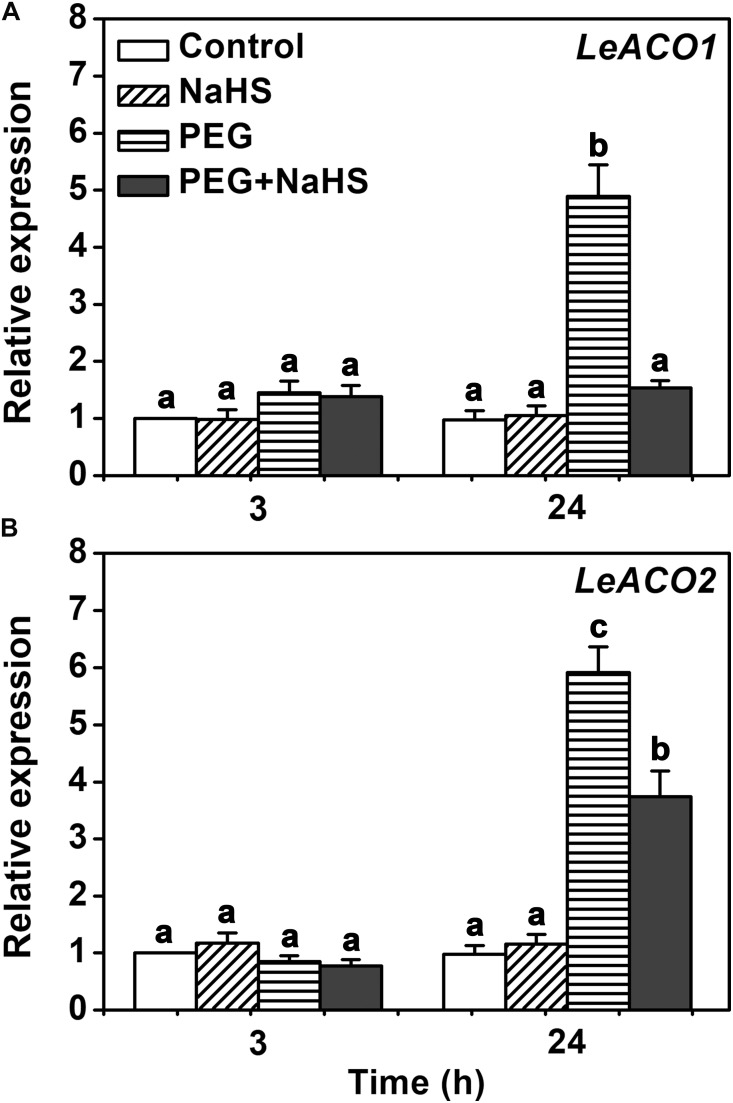
The expressions of *LeACO1* and *LeACO2* genes analysis in leaves. **(A)** Relative expression of *LeACO1*. **(B)** Relative expression of *LeACO2*. Two-week-old tomato seedlings were supplied with 25 % PEG, 200 μM NaHS and 1.0 g/L Ethephon for 3 h or 24 h, and qRT-PCR used for gene expression analysis. Within each set of experiments, bars with different letters are significant different (Duncan’s multiple range tests, *P* < 0.05).

To investigate in how far H_2_S influences the transcription of ACC oxidase, the relative expressions of *LeACO1* and *LeACO2* were analyzed in tomato leaves. Quantitative real-time PCR showed that the gene expressions of *LeACO1* and *LeACO2* were not changed by NaHS or osmotic stress at 3 h (**Figure 6**). However, the gene expressions of *LeACO1* and *LeACO2* were markedly induced after 24 h exposure to osmotic stress. Interestingly, this increase was attenuated when NaHS was supplied to the stressed plants (**Figure 6**). The results suggest that H_2_S does not directly affects ACC oxidase expression.

## Discussion

### H_2_S Is Required for Ethylene-Induced Stomatal Closure

The data of García-Mata and Lamattina (2010) has provided evidences that H_2_S could induce the stomatal closure of *Arabidopsis*, *Vicia faba* and *Impatiens walleriana*. Here, we show that these results also apply to tomato (**Figure 1**). It is interesting that H_2_S signal often interacts with phytohormone or signaling molecules to regulate many physiological processes (Hou et al., 2013; Scuffi et al., 2014; Jia et al., 2015). Recently, H_2_S has been proven to participate in ABA- or ETH-induced stomatal closure in *Arabidopsis*. (García-Mata and Lamattina, 2010; Liu et al., 2011). ABA or ethylene can regulate H_2_S generation. H_2_S production in tomato is shown to be induced by ethylene similar to what was observed in *Arabidopsis* and *V. faba* (**Figure 2A**; Liu et al., 2011, 2012). Furtherly, our results provided details of how H_2_S signaling and ethylene signaling act together in the osmotic stress response. Importantly, the stomatal opening in response to osmotic stress is shown to depend on H_2_S signaling as it could be prevented by application of a scavenger or inhibitor of H_2_S synthesis. Pharmacological studies also showed that ethylene is insufficient to induce stomatal closure when H_2_S action is inhibited (**Figure 2E**). These results strongly suggested that H_2_S is an important signal molecule involved in ethylene signal, at least in the osmotic stress response of guard cells. Furthermore, results confirmed that H_2_S signaling generally interacts with phytohormone signaling to regulate the physiological processes (Hou et al., 2013; Scuffi et al., 2014; Jia et al., 2015).

### H_2_S Controls Feedback Regulation of Ethylene Biosynthesis Through Persulfidation of LeACO1 and LeACO2

1-aminocyclopropane-1-carboxylic acid oxidases regulate the last, rate-limiting step of ethylene biosynthesis (Argueso et al., 2007). The activity of ACOs was shown to directly affect the endogenous ethylene content (Groen and Whiteman, 2014). It was reported that the activity of ACOs is regulated by plant signaling molecules, such as nitric oxide (Tierney et al., 2005; Zhu et al., 2006). Recently, it was suggested that H_2_S antagonizes the effect of ethylene action in banana fruit ripening and senescence (Ge et al., 2017). Therefore, H_2_S may resist the function of ETH. In this work, we found that H_2_S inhibited the ETH generation under osmotic stress (**Figure 4**). In addition, the activity of ACO also is inhibited by H_2_S in tomato leave which is similar to nitric oxide (Zhu et al., 2006).

In this work, we found that H_2_S inhibited the ethylene generation under osmotic stress condition, presumably due to the inhibition of ACO activity (**Figure 3**). This regulation was shown here to work via persulfidation of one specific cysteine residue of the LeACO proteins (**Figures 4**, **5**). Negative enzyme activity regulation by H_2_S-induced persulfidation has been previously reported for the protein tyrosine phosphatase PTP1B (Krishnan et al., 2011) and a phosphodiesterase (Bucci and Cirino, 2011). In addition, LeACO1^C60S^ was insensitive to NaHS treatment comparison with LeACO1^WT^, suggesting that the Cys60 is the target site of H_2_S-induced persulfidation.

### H_2_S Inhibits the Transcription of Ethylene Biosynthesis Genes Through an Indirect Way

Transcriptional regulation of ethylene biosynthesis genes is an important way for modulating ethylene signaling. Phytohormone, signaling molecules and environment stress can change the transcription of these genes, including ethylene itself (Wang et al., 2002). Our data showed that H_2_S did not alter the expressions of *LeACOs* within 3 h (**Figure 6**), suggesting that H_2_S did not directly affect the transcriptions of *LeACOs*. While expression of *LeACO1* and *LeACO2* under osmotic stress at the late time point (24 h) was reduced in H_2_S-treated plants compared to control plants, this is likely to be an indirect effect of the H_2_S-induced inhibition of ACO activity. This reduces ethylene content and, thereby, reduce the positive regulation of ethylene biosynthesis by ethylene itself that has been documented previously (Jakubowicz et al., 2010).

## Conclusion

**FIGURE 7:**
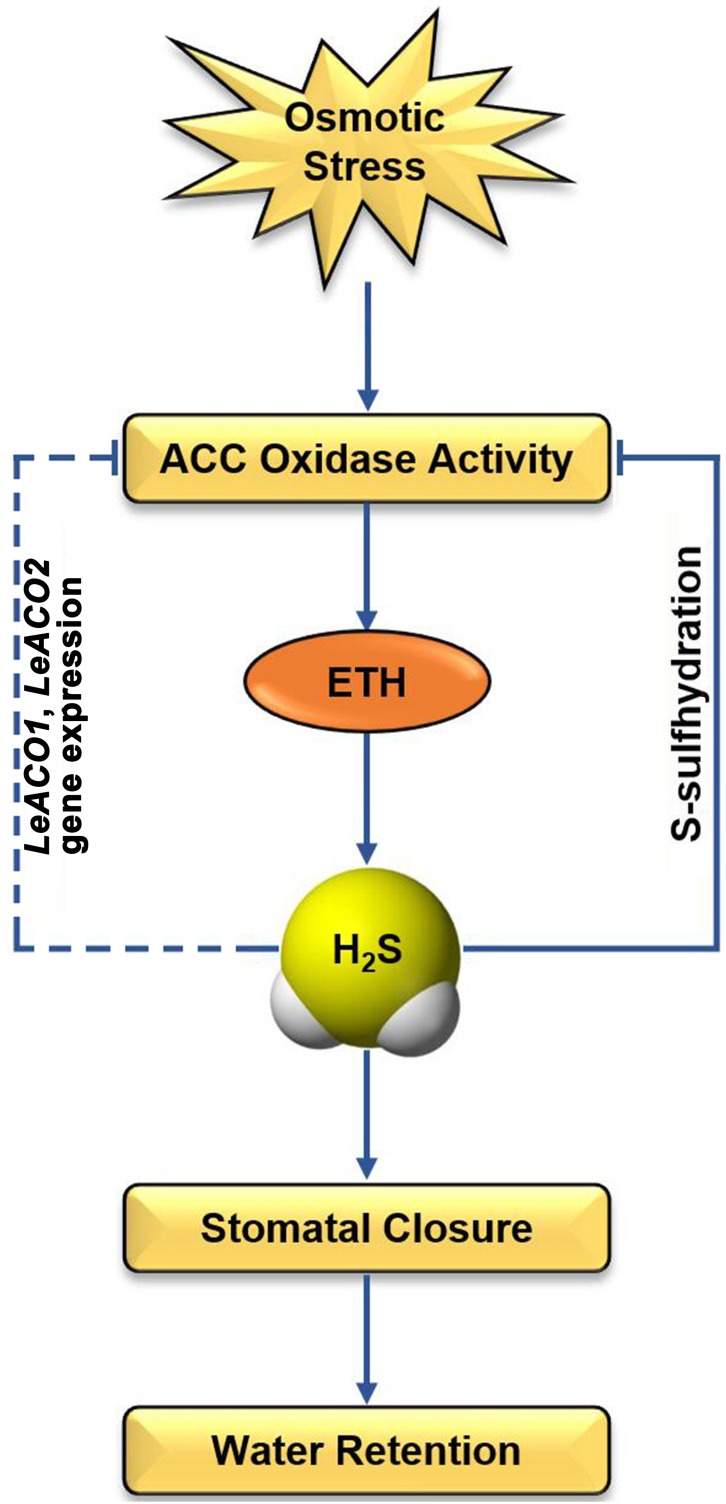
Schematic diagram illustrating the proposed signaling regulation of ethylene-induced H_2_S negatively regulates ethylene biosynthesis in tomato. Arrows indicate enhanced effects and hyphens indicate suppressed effects.

ETH, as a stress phytohormone, generates under environmental stress and enhances the resistance of plants (Bleecker and Kende, 2000). However, the excessive accumulation of ETH led to cell senescence and programmed cell death (Lacey and Binder, 2014). Therefore, the regulation of ETH biosynthetic is important for plant under environmental stress. Our study revealed a novel mechanism that is H_2_S feedback regulates ETH biosynthesis in tomato under osmotic stress (**Figure 7**). Our evidences provided that ETH enhances the accumulation of H_2_S in guard cells. H_2_S plays the dual role ethylene-induced stomatal closure. On the one hand, ETH-induced H_2_S is a required for ETH-induced stomatal closure. On the other hand, ethylene-induced H_2_S negatively regulates ethylene biosynthesis through persulfidation of LeACO1 and LeACO2. Post-translational modification of ACOs maybe a direct way of H_2_S regulates ETH biosynthesis. In addition, H_2_S also inhibits the transcription of ETH biosynthesis genes through an indirect way. Overall, this study presents compelling evidence supporting interaction between ethylene and H_2_S and these results are important in context of the biotechnological improvement of osmotic stress tolerant tomato plants.

## Author Contributions

JiL and HJ designed and performed the research. SC, JY, CS, XW, and WS screened the mutant plants. HJ, SC, DL, JW, MR, CS, XW and JY analyzed the data. JoL edited the language. JiL wrote the paper.

## Conflict of Interest Statement

The authors declare that the research was conducted in the absence of any commercial or financial relationships that could be construed as a potential conflict of interest.

## Footnotes

1 rsb.info.nih.gov/ij/
