# Supplementary material for: Ethylene-Induced Hydrogen Sulfide Negatively Regulates Ethylene Biosynthesis by Persulfidation of ACO in Tomato Under Osmotic Stress
Source: Front Plant Sci. 2018 Oct 17;9:1517. doi: 10.3389/fpls.2018.01517 (PMC6199894; doi:10.3389/fpls.2018.01517)
Supplement: Supplementary file 1 [file Table_1.DOC]

**Supplemental Table**

**Table S1** Primers used in this study.

|  |  | Accession number | Primer 5’-3’ | Sequence 5’-3’ |
| --- | --- | --- | --- | --- |
| 1 | *Real-time PCR* |  | *Ubi3-qPCR-F* | CGTGGTGCTAAGAAGAGAGGAAG |
| 2 |  |  | *Ubi3-qPCR-R* | GAACTGCAACACAGCGAGCTTAAC |
| 3 |  | NM_001247095.2 | *LeACO1-qPCR-F* | GGTTACTTGAAAAATGCCTTTTA |
| 4 |  |  | *LeACO1-qPCR-R* | GTCTTTGAGGAGTTGAAGGCCA |
| 5 |  | NM_001329913.1 | *LeACO2-qPCR-F* | ATGGAACAAAGATTTAAAGAATT |
| 6 |  |  | *LeACO2-qPCR-R* | TCTTTTAGCAAAATCCCTCATA |
| 7 | *Protein Expression* | NM_001247095.2 | *LeACO1-ex-F* | ATGGAGAACTTCCCAATTAT |
| 8 |  |  | *LeACO1-ex-R* | CTAAGCACTTGCAATTGGAT |
| 9 |  | NM_001329913.1 | *LeACO2-ex-F* | ATGGAGAATTTCCCAATTAT |
| 10 |  |  | *LeACO2-ex-R* | CTAAGCAATTGCAATTGGAT |
